# Can a powered knee-ankle prosthesis improve weight-bearing symmetry during stand-to-sit transitions in individuals with above-knee amputations?

**DOI:** 10.1186/s12984-023-01177-w

**Published:** 2023-05-02

**Authors:** Grace R. Hunt, Sarah Hood, Lukas Gabert, Tommaso Lenzi

**Affiliations:** 1 Department of Mechanical Engineering, University of Utah, Salt Lake City, UT 84112 USA; 2 Rocky Mountain Center for Occupational and Environmental Health, Salt Lake City, UT USA

**Keywords:** Powered prosthetics, Sit-down, Transfemoral, Amputee, Wearable robotics, Knee-ankle prosthesis

## Abstract

**Background:**

After above-knee amputation, the missing biological knee and ankle are replaced with passive prosthetic devices. Passive prostheses are able to dissipate limited amounts of energy using resistive damper systems during “negative energy” tasks like sit-down. However, passive prosthetic knees are not able to provide high levels of resistance at the end of the sit-down movement when the knee is flexed, and users need the most support. Consequently, users are forced to over-compensate with their upper body, residual hip, and intact leg, and/or sit down with a ballistic and uncontrolled movement. Powered prostheses have the potential to solve this problem. Powered prosthetic joints are controlled by motors, which can produce higher levels of resistance at a larger range of joint positions than passive damper systems. Therefore, powered prostheses have the potential to make sitting down more controlled and less difficult for above-knee amputees, improving their functional mobility.

**Methods:**

Ten individuals with above-knee amputations sat down using their prescribed passive prosthesis and a research powered knee-ankle prosthesis. Subjects performed three sit-downs with each prosthesis while we recorded joint angles, forces, and muscle activity from the intact quadricep muscle. Our main outcome measures were weight-bearing symmetry and muscle effort of the intact quadricep muscle. We performed paired t-tests on these outcome measures to test for significant differences between passive and powered prostheses.

**Results:**

We found that the average weight-bearing symmetry improved by 42.1% when subjects sat down with the powered prosthesis compared to their passive prostheses. This difference was significant (p = 0.0012), and every subject’s weight-bearing symmetry improved when using the powered prosthesis. Although the intact quadricep muscle contraction differed in shape, neither the integral nor the peak of the signal was significantly different between conditions (integral p > 0.01, peak p > 0.01).

**Conclusions:**

In this study, we found that a powered knee-ankle prosthesis significantly improved weight-bearing symmetry during sit-down compared to passive prostheses. However, we did not observe a corresponding decrease in intact-limb muscle effort. These results indicate that powered prosthetic devices have the potential to improve balance during sit-down for individuals with above-knee amputation and provide insight for future development of powered prosthetics.

## Background

The ability to safely and comfortably stand up and sit down is critical for daily life. Healthy adults stand up and sit down approximately 60 times per day [1]. Standing up and sitting down are related tasks, which require similar amounts of joint torque. However, standing up is primarily a concentric muscle activity, meaning that the leg muscles contract while shortening and perform positive work as the body’s mass is lifted. In contrast, sitting down is an eccentric muscle activity, meaning that the leg muscles contract while lengthening and perform negative work as the body’s mass is lowered. During both stand-up and sit-down, the user must also control their center of mass in order to maintain dynamic balance [2], [3]. Sitting down is generally performed with more caution than standing up, because there is less visual information and, therefore, less certainty about the location of the movement’s endpoint (i.e., the chair) [2]. Sitting down is especially difficult for many individuals with physical disabilities due to reduced motor coordination, strength, and/or balance. In populations with impaired mobility, the initiation of sit-down is often followed by a rapid, uncontrolled descent into the chair [4]. Sitting down without sufficient control is both dangerous and uncomfortable and can result in injury or falls. In individuals with physical disabilities, including lower limb amputees, fear of falling is often associated with self-imposed restriction of activities, which can, itself, lead to reduced balance, strength, and coordination [5]. Sitting down is an essential daily activity, which should not be overlooked during rehabilitation for populations with mobility impairments.

Sitting down in a controlled and safe manner is difficult for above-knee amputees. After above-knee amputation, the missing biological knee and ankle are typically replaced with passive prosthetic devices. Passive prosthetics use dampers to provide resistance and support during movement. Microprocessor knees are the most commonly prescribed type of prosthetic knees. Microprocessor knees are energetically passive, meaning they cannot perform positive work or provide positive energy. However, they have miniaturized actuators that can actively adjust the level of resistance provided by the prosthetic joint. For example, in some microprocessor knees, the level of damping can be changed during or between activities by opening and closing valve of a hydraulic damper system [6–8]. In microprocessor-controlled knees, the controlled flexion of the knee joint is commonly referred to as “yielding”. “Yielding” provides variable joint resistance during the stance phase of walking, stair descent, and sit-down. Although the microprocessor can increase resistance during the sitting down movement, there is a limit to the resistance the dampers in the knee can provide. Most microprocessor knees use linear hydraulic or pneumatic systems, which are connected to the knee joint via four-bar linkages [6–8]. These four-bar linkages have geometric singularities which occur at around 90° of knee flexion. When a microprocessor knee reaches its geometric singularity, the damper cannot provide resistance, and the knee joint acts like a simple hinge. Thus, microprocessor-controlled knee prostheses cannot provide resistance to the knee joint at the end of the sitting down movement when users need the most support. Moreover, due to the nature of their damping systems, the resistive torque provided by passive knee prostheses is proportional to the knee velocity and, therefore, cannot be precisely controlled. Because microprocessor knees cannot provide adequate support during essential parts of the movement or provide high levels of assistance when needed, these prosthetic devices fail to adequately perform the support functions of a biological leg. Partly due to these design limitations, prosthesis users either over-compensate with their intact leg, residual limb, trunk, and upper body, or sit down with faster, less controlled, and less comfortable movements than able-bodied persons [9]. Compensatory movements can exacerbate secondary conditions such as back pain and osteoarthritis, while increasing the risk of injury and falls [10]. Thus, there is an urgent and unmet need for leg prostheses that can improve stand-to-sit transitions.

Powered prostheses have the potential to improve the mobility of amputees. Different from conventional passive prostheses, powered prostheses can perform positive work and provide positive energy using motors and batteries [11]. Because of this unique ability, most powered prosthetic research has focused on activities that require performing positive work such as standing up, climbing stairs, and walking [12], [13], [22–25], [14–21]. In contrast, activities that require performing only negative work have received much less attention [12], [14], [19], [23], [26], [27]. However, powered prostheses have the potential to improve negative work tasks as well. Powered prosthetics can produce higher levels of resistance compared to passive prostheses, especially at higher knee flexion angles. Moreover, powered prostheses can more accurately control the torque provided by the prosthetic joint at each point in a movement. For example, a powered prosthesis can control the resistive torque during sit-down as a function of the knee joint position to provide a more physiological torque profile [28], [29], or as a function of residual muscle activation to give users direct control of the prothesis resistance [12]. Thus, powered prostheses have the potential to give above-knee amputees more control and support during the sit-down movement than passive prostheses.

Only one powered knee prosthesis is widely available on the market, the Ossur POWER KNEE. A case study with one subject performing 3 sit-down movements showed that the Ossur POWER KNEE improved weight-bearing symmetry compared to the Ottobock C-Leg, a passive microprocessor knee [30]. However, these results were not reproduced in a larger study comparing 7 subjects in each of 4 groups: the Ossur POWER KNEE, a powered knee prosthesis; the Mauch SNS, a passive knee prosthesis with a fixed level of hydraulic damping; the C-leg, a passive knee prosthesis with variable damping; and a healthy, non-amputee control group. The POWER KNEE improved weight-bearing symmetry significantly compared to the Mauch SNS knee, but not compared to the C-Leg. Interestingly, the inverse dynamics analysis shows that the Mauch SNS produced a small flexion torque on average, rather than a supportive extension torque – it did not provide resistance to slow down the stand-to-sit movement. In contrast, both the POWER KNEE and C-Leg produced extension torque to slow the user during the stand-to-sit transition. In fact, the POWER KNEE and C-Leg provided similar levels of peak knee extension torque, which was still only about 1/8 the magnitude of the healthy control group’s peak knee torques [31]. This study shows that the POWER KNEE does not produce higher levels of knee resistance compared to the C-Leg and fails to improve weight-bearing symmetry. However, this result may be due to limitations of the POWER KNEE and may not generalize to other powered knee prostheses. Therefore, the potential of powered knee prosthesis to increase knee resistance and control stand-to-sit transitions has not been realized by market-available powered knee prostheses.

Research powered prostheses provide a scientific tool to understand how the assistance or resistance provided at the prosthesis joints affect user performance during different tasks, including stand-to-sit transitions. Sit-down with research powered devices has been demonstrated using different devices and control algorithms [12], [19], [28], [29], although comparisons with passive prostheses are generally lacking. One study with seven individuals with above-knee amputations showed that a research powered knee-ankle prosthesis supported up to 47% more force during sit-down than a microprocessor-controlled prosthesis [32]. Notably, in this study the powered prosthesis was programmed to imitate microprocessor-controlled prostheses using a virtual damping control [32]. It is not clear how the powered prosthesis achieved this result because motion capture was not used, inverse dynamics were not performed, and time-domain plots were not shown [32]. Thus, powered prostheses have shown the ability to improve weight-bearing symmetry during sit-down compared to microprocessor-controlled knees, but the reasons for this improvement are not known.

The primary objective of this study is to determine whether a powered knee-ankle prosthesis can improve sit-down performance in above-knee amputees. We hypothesize that the powered prosthesis can improve weight-bearing symmetry and muscle effort during sit-down compared to passive prostheses by providing higher resistance at the prosthetic knee joint. The secondary objective of this study is to assess how powered prosthetics impact the sit-down movement compared to passive prostheses to identify critical factors that may enable future improvements. Specifically, we aim to assess weight-bearing symmetry, joint kinetics, joint kinematics, and joint energy injection or dissipation during the sit-down movement. To accomplish these goals, we recruited ten individuals with above-knee amputations. The subjects sat down using their prescribed passive prosthesis and a research powered prosthesis [33] while we recorded joint angles, ground reaction forces, and muscle activity from the intact quadricep muscle. As primary outcomes to test our hypothesis, weight-bearing symmetry was measured using the average index of asymmetry of the vertical ground reaction force, and muscle effort was measured using the peak and integral of the intact vastus medialis muscle EMG. By providing the first full biomechanical analysis of above-knee amputees sitting-down with powered prostheses, this study lays the foundation for future clinical use and continued development of powered prostheses.

## Methods

### Participants

Ten individuals with above-knee amputations participated in this study. Inclusion criteria were unilateral above-knee amputation, daily use of prescribed prosthesis, and ability to sit down into a chair with or without the use of hands. Exclusion criteria included any musculoskeletal, cardiovascular, or other impairments that would prevent a subject from completing the study activities. More details on the subjects can be found in Table I. The study protocol was approved by the University of Utah’s Institutional Review Board (Protocol 00103197, approved 6/16/2021). Subjects provided written informed consent before the experiment began, including consent to publish photographs and videos of the experiments. A certified prosthetist was present during all experiments.

**Table I:** Subject information

Subject Code	Sex	Age [years]	Weight [kg]	Height [m]	Age of Amputation [years]	Amputation Side	Reason for Amputation	Previous Experience with Utah Bionic Leg	Passive Knee Prosthesis	Passive Ankle Prosthesis	Socket Suspension	Used hands during sit-down	Desired peak knee extension torque [Nm/kg]
TF01	M	64	88.64	1.880	5	L	Trauma	N	Rheo	Proflex XC	Vacuum	N	0.3
TF02	M	52	102.27	1.905	13	L	Trauma	Y*	C-Leg	Triton	Pin Lock	N	0.2
TF03	M	29	65.14	1.778	8	R	Trauma	Y	Plie	All Pro	Suction	N	0.3
TF04	F	32	59.09	1.600	12	L	Trauma	Y*	Plie	All Pro	Lanyard	N	0.5
TF05	F	60	58.64	1.651	13	L	Trauma	N	C-Leg	Trias	Suction	N	0.2
TF06	M	40	90.45	1.905	35	L	Trauma	Y	Plie	Soleus	Suction	N	0.6
TF07	M	38	100.45	1.803	10	L	Trauma	N	C-Leg	All Pro	Suction	N	0.2
TF08	M	53	99.8	1.930	22	L	Trauma	N	C-Leg	Taleo	Suction	Y	0.4
TF09	F	26	68.18	1.753	7	R	Trauma	N	Plie	Proflex XC	Suction	Y	0.6
TF10	M	63	86.82	1.702	5	L	Dysvascular	Y	C-Leg	Kinterra F1	Lanyard	Y	0.15

* asterisks = subject has used the previous version of the Utah Bionic leg, but had not used the current version before participating in this study

### Instrumentation

**Fig. 1:**
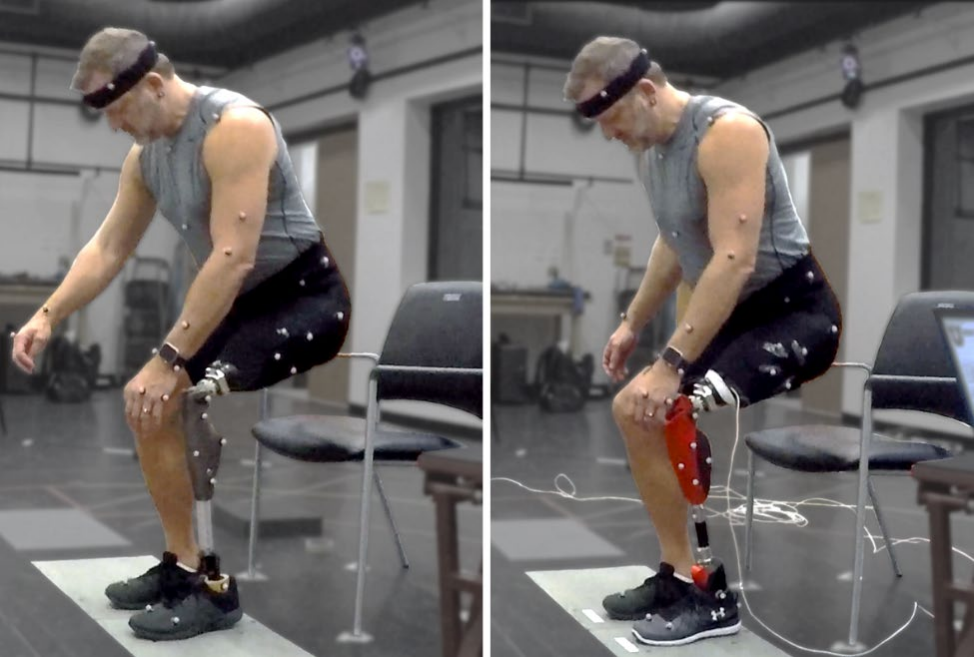
A subject sitting down with passive (left) and powered (right) prostheses during the experiment. Retroreflective markers on the subject track the movement of their body segments, and separate six-axis force plates record ground reaction forces from each leg. A wire connected to the powered leg synchronized data recording

We asked study participants to dress in tight-fitting clothing [Fig. 1], and we placed retroreflective markers (14 mm diameter, Vicon Motion Systems, Centennial, CO, USA) on their bony prominences and other landmarks. We followed a modified plug-in-gait marker set to track 15 segments (2 feet, 2 shanks, 2 thighs, pelvis, trunk, head, 2 arms, 2 forearms, 2 hands). We placed markers for the prosthesis ankle joint axis proximal to flexible ankle components of the passive ankles, and lateral to the rotary axis of the powered ankle joint. A 12-camera Vicon motion capture system captured the 3D location of the retroreflective markers at 200 Hz. Two AMTI OR6-7 biomechanics platforms (Advanced Medical Technology, Watertown, MA) recorded ground reaction forces from under each foot at 1000 Hz. We captured a static trial with each prosthesis (prescribed passive prosthesis and research powered prosthesis) while subjects stood in the recommended static calibration pose (Vicon Nexus 2.1, Vicon Motion Systems, Centennial, CO). We captured a functional range of motion trial with each prosthesis in order to calibrate functional joint centers [35, 36]. We recorded electromyography (EMG) signals from the vastus medialis muscle of the intact leg. We located the vastus medialis following SENIAM placement procedures [36]. We shaved the skin over the muscle, wiped the skin with alcohol, and attached a Delsys Tringo Avanti EMG sensor to the prepared skin using an adhesive. The EMG system was time-synchronized and recorded by Vicon Nexus.

**Fig. 2:**
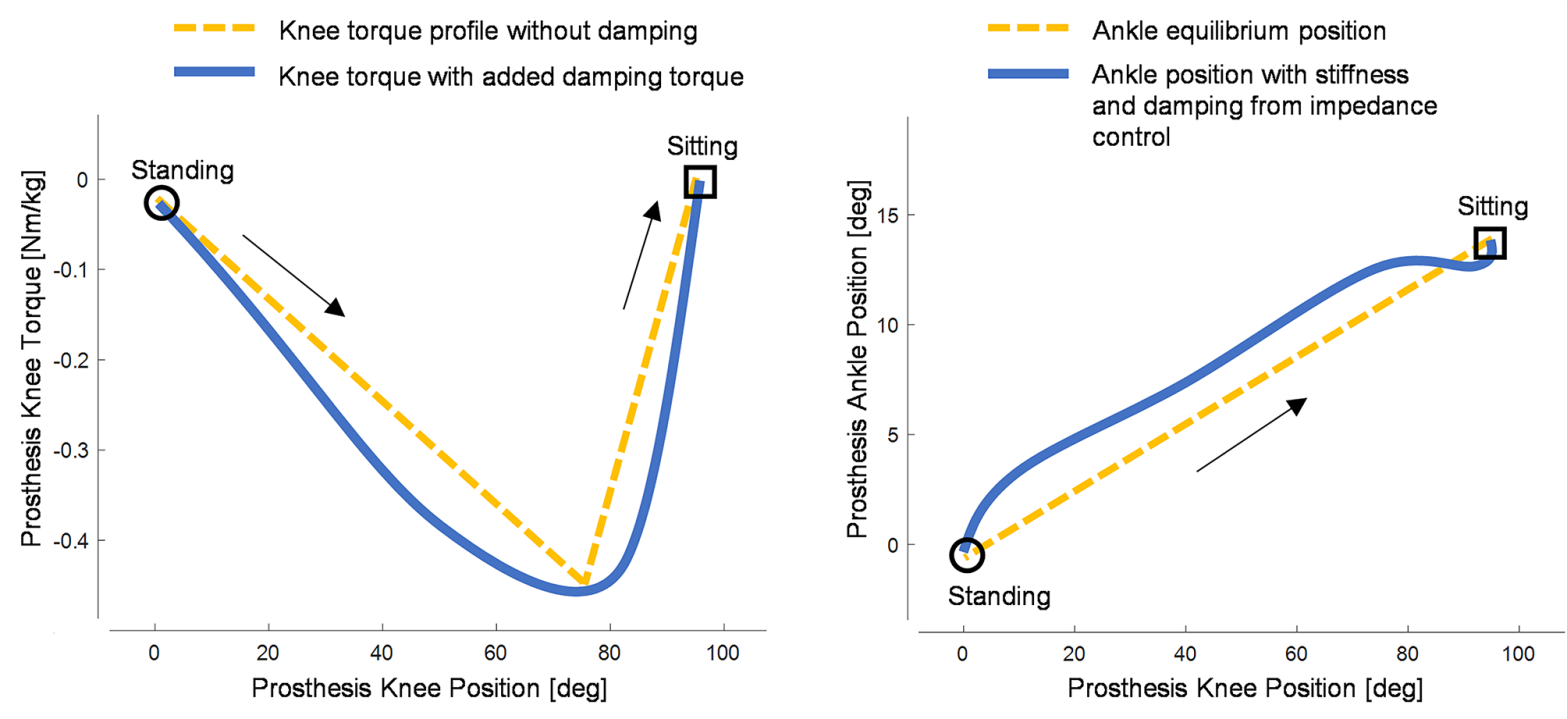
Sit-down controller. Left: Prosthesis knee torque was controlled as a function of prosthesis knee position. A triangle-shaped profile was prescribed (yellow), and damping torque was added (blue). Right: prosthesis ankle equilibrium position was controlled as a function of prosthesis knee position (yellow). The equilibrium position was used in an impedance controller that added damping and stiffness around the equilibrium position, resulting in the prosthesis ankle position (blue). The start of the movement (standing) is indicated with circles, and the end of the movement (sitting) is indicated with squares. Black arrows indicate the progression of the movement

### Powered prosthesis

The powered prosthesis used in this study was the Utah Bionic Leg, a battery-powered, lightweight robotic prosthesis with knee and ankle/foot modules. Both the knee and ankle can provide up to 150 Nm of torque in flexion or extension during movements. The prosthesis, including the powered knee module, powered ankle/foot module, batteries, and protective covers, weighs 3.2 kg [33]. The powered knee module uses a novel torque-sensitive actuator. The torque-sensitive actuator combines the benefits of a variable transmission with that of a series elastic actuator. A passive mechanism actuated by a spring reacts to the knee extension torque by increasing the moment arm of the linear actuator powering the knee. As a result, the transmission ratio changes passively, continuously, and quickly in response to varying knee extension torque. This actuation mechanism allows a small motor to efficiently provide the wide ranges of torque and speed required at the knee during ambulation. The powered ankle/foot module has joints at the ankle and toe which are both actuated by a single actuator [33]. A custom instrumented pyramid adapter was attached to the top of the ankle module to estimate the vertical ground reaction force and sagittal plane torques [37, 38]. A certified prosthetist cut a standard aluminum pylon to the appropriate height for each subject, and the pylon was used to connect the knee module to the ankle/foot module. Wires connected the electronics of the knee and ankle/foot to synchronize movements. The powered prosthesis’s high-level controller uses finite-state machines to define the prosthesis behavior for each activity [39]. The sit-down controller is described in the next section. The middle-level controller uses control algorithms to generate desired joint torque or joint position. The low-level controller translates the desired joint torque or position into current commands, which are sent to the motors of the knee and ankle/foot modules.

### Powered prosthesis sit-down controller

During sit-down, the assistive knee torque was defined as the combination of two components. The first component was a triangular-shaped torque profile, resulting in a resistive torque that was dependent on the knee joint position (Fig. 2, left, yellow), which cannot be replicated by passive prostheses. The second component was a virtual damping torque, which provided a resistive torque that was proportional to the knee velocity, similar to the function of passive prostheses. The triangular-shaped torque profile was defined using three points in the torque/position plane – a point at the start of the movement, a point defining the torque and position at peak torque, and a point at the end of the movement, shown in (Fig. 2, left, yellow). The torque-position profile was inspired by the biomechanics of the nonamputee sit-down motion [40]. At the beginning of the sit-down movement, when the knee prosthesis was at the maximum extension (i.e., knee joint angle close to zero), the knee provided a very small extension torque (-2.5 Nm, approximately -0.03 Nm/kg) to keep the knee from collapsing before the subject was ready to sit. As the knee started flexing (increasing the knee joint angle), the desired resistive torque increased to its maximum extension torque (negative). Finally, the knee torque approached zero torque when the knee was fully flexed (positive knee joint angle) and the sit-down motion was completed. The desired knee joint position at which the peak torque was reached was set in the controller software to approximately 85% of each subject’s sitting knee angle, similar to able-bodied sit-down biomechanics [40]. The powered ankle joint was controlled using impedance control, using fixed virtual stiffness (K = 3 Nm/deg) and fixed virtual damping (B = 0.2 Nm/deg/s) similar to our previous work [12, 13]. The ankle equilibrium position was controlled as a function of knee position (Fig. 2, right, yellow). At the beginning of sit-down, the knee is extended (near zero), and the ankle is neutral (near zero). At the end of sit-down, the knee is flexed and the ankle is dorsiflexed. The ankle equilibrium angle was changed linearly between these two points. The ending point of the knee-ankle relationship was adjusted so that each subject’s foot was flat on the ground during sitting. The stiffness and damping added by impedance control added some flexibility about the prescribed equilibrium position (Fig. 2, right, blue).

### Experimental protocol

All test procedures were performed using each subject’s prescribed, passive prosthesis (“passive” trials), and then repeated with the research powered prosthesis, the Utah Bionic Leg (“powered” trials). We asked subjects to keep their feet equally placed on two force plates, and we marked their foot positions with tape. We asked subjects to pause between each stand up and sit-down movement. If needed, we corrected their foot positions between each movement. We asked subjects to sit down without using their hands if possible, and seven subjects were able to do this (TF01, TF02, TF03, TF04, TF05, TF06, TF07). We asked the three subjects who needed to use their hands for support during passive sit-down (TF08, TF09, TF10) to use their hands during sit-down with both prostheses. We could not calculate accurate inverse dynamics (joint torques and joint change in energy) for the three subjects who sat down using hands because the chair’s armrests were not instrumented, so we excluded these subjects from plots and metrics that required inverse dynamics. TF07’s EMG recording was unusable because of movement artifact, so we eliminated this subject from EMG plots, metrics, and statistics.

After donning the Utah Bionic Leg, subjects performed several practice sit-downs with increasing levels of peak assistive torque. We had initially planned to apply 0.93 Nm/kg peak torque based on able-body reference data [40]. However, during the experiments we found that subjects had difficulty sitting down with this level of assistance. Specifically, subjects had issues flexing the knee joint without performing abnormal movements such as twisting their trunk. To address this issue, for each subject, we increased assistance until we found the highest level of resistance that enabled each subject to sit down without difficulty or abnormal movements. Each subject’s desired peak torque level is reported in Table I. Finding each subject’s level of peak sit-down torque assistance took between 5 and 10 min. The subjects rested in between stand-to-sit transitions with different peak assistances. After we determined the peak knee torque each subject felt comfortable sitting down with, the subjects rested until they were ready to continue (approximately 5–10 min), and then performed 5–8 powered sit-down movements which were recorded.

### Data processing

We recorded, synchronized, and processed marker trajectories and force plate data using Vicon Nexus (Vicon Nexus 2.1). We calculated hip joint centers using symmetrical center of rotation estimation [35], and knee joint axes using symmetrical axis of rotation estimation [34]. We transferred the data to Visual 3D (C-Motion, Germantown, USA). We filtered the marker trajectories and force plate data using bidirectional, fourth-order Butterworth filters with cutoffs of 6 and 15 Hz, respectively. We determined these cutoff frequencies using residual analysis [41]. We used Visual 3D to create biomechanical models of each subject’s body segments. We modified the model for the prosthesis shank following [42, 43]. We calculated joint angles and joint moments for the hip, knee, and ankle joints. We imported synchronized joint data, recordings from the Utah Bionic Leg, and EMG recordings into MATLAB for processing (Mathworks, Natick, MA). We created EMG “envelopes” from the raw EMG recordings by bandpass filtering between 20 and 450 Hz, rectifying, and filtering again using a low pass, fourth order, bidirectional Butterworth filter with a 3 Hz cutoff frequency. We calculated the index of asymmetry at each point in time using Eq. 1 after [30, 44, 45].

1 $$IOA = \frac{{Intact{\text{ }}GRF - Prosthesis{\text{ }}GRF}}{{Intact{\text{ }}GRF + Prosthesis{\text{ }}GRF}}*100$$

The index of asymmetry can also be calculated by subtracting the intact-side GRF from the prosthesis-side GRF, which reverses the sign. We calculated secondary variables (velocities, powers, etc.). We filtered all signals using a bidirectional, fourth-order Butterworth filter with a 3 Hz cutoff frequency. Sit-down movements were segmented based on prosthesis knee position and prosthesis knee velocity. We segmented each sit-down movement iteration and interpolated each iteration to the same length from 0 to 100% of sit-down. We also segmented and interpolated from 0 to 120% of sit-down, to ensure that all signals of interest during the entire movement were included for visualization purposes. We eliminated sit-down iterations with problems, and then selected the last 3 of the remaining iterations for each subject for analysis. We selected three repetitions because every subject performed at least three repetitions without EMG artifacts or other problems, and used the last three because they were performed after the most practice. We normalized each subject’s powered and passive EMG envelopes by the peak of the time-average of the subject’s three passive trial EMG envelopes.

We created time-plots by averaging the interpolated signals from each subject’s 3 sit-down iterations, resulting in a single mean signal for each subject. We used the individual subject means to calculate across-subject means and standard errors which are shown in all time plots. We derived all torque-based plots and metrics using data from the 7 subjects who did not use hands during sit-down. We derived all non-torque-based plots and metrics using data from all 10 subjects, with the exception of the EMG plot [Fig. 4] and related EMG metrics and statistics, which do not include TF07 because of EMG movement artifact.

We calculated metrics for each subject’s 3 sit-down iterations, then averaged them to find single-subject means. We used the single-subject means to calculate across-subject means and standard errors. For each iteration, we quantified weight-bearing asymmetry by averaging the index of asymmetry at each point in time between 0 and 100% of sit-down completion. For each iteration, we quantified muscle effort of the intact vastus medialis by finding the peak and integral (calculated with non-normalized time) of the normalized EMG envelope between 0 and 100% of sit-down completion. For each iteration, we calculated all other metrics between 0 and 120% of sit-down completion. We averaged the metric from each subject’s 3 iterations to calculate single-subject means. We averaged the single-subject means to calculate across-subject means and standard errors. All metrics in the body of the paper are across-subject means and standard errors, presented as mean ± standard error. The height of each bar in a bar plot represents an across-subject mean. Error bars indicate the standard error of the across-subject mean. The single-subject means are shown on bar plots for both passive and powered conditions with colored dots, connected with colored lines.

**Fig. 3:**
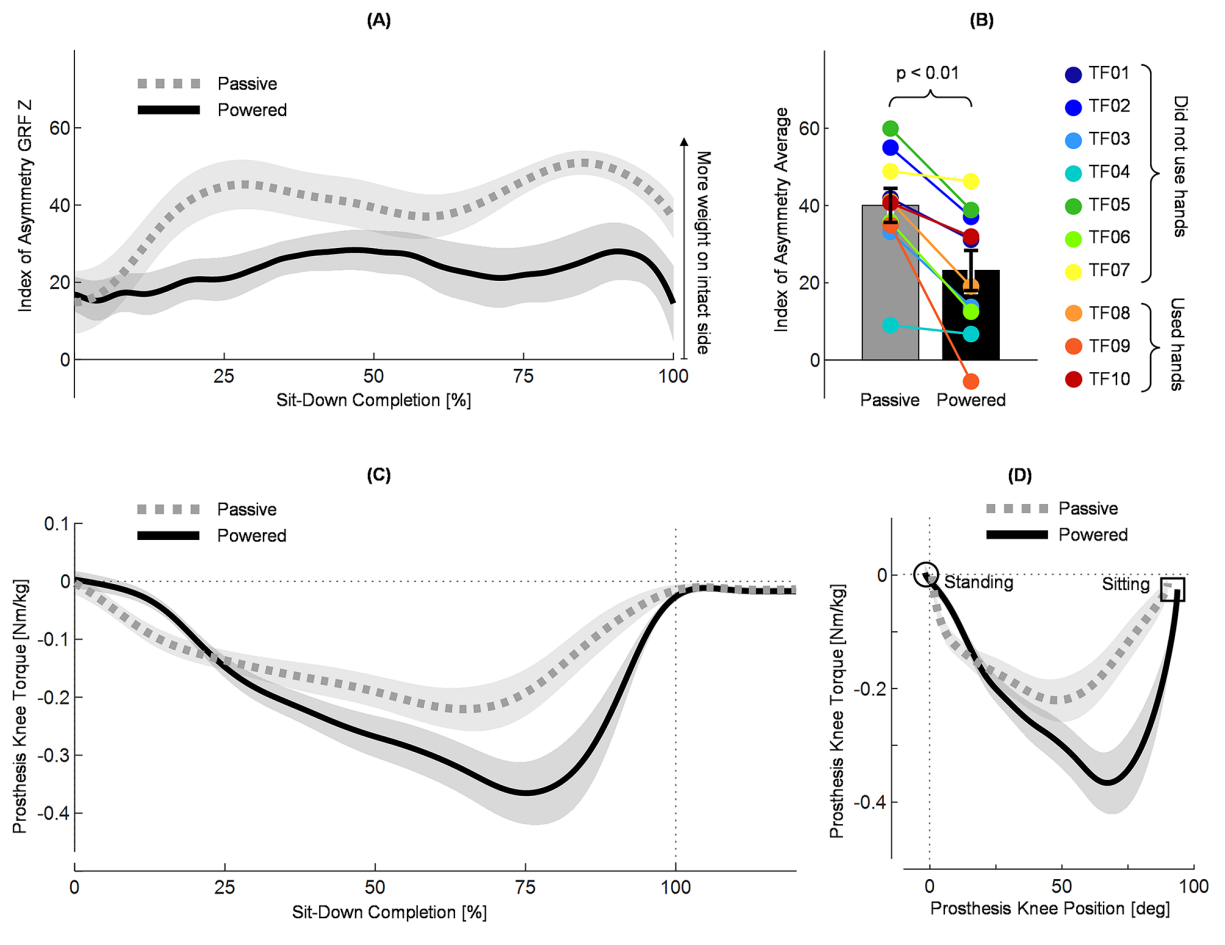
Weight-bearing asymmetry and prosthetic knee torque. (A) Index of Asymmetry (IOA) of the vertical GRF during sit-down with passive (gray) and powered (black) prostheses for all subjects from 0 to 100% of sit-down completion. Negative IOA indicates more weight on the prosthesis; positive IOA indicates more weight on the intact leg. Lines indicate across-subject means and shading indicates standard error (N=10 subjects). (B) Average IOA calculated between 0 and 100% of sit-down completion. Bar heights indicate the across-subject mean IOA (the across-subject average of the single-subject IOA means), and error bars indicate standard error (N=10 subjects). A paired t-test compared the across-subject means for passive and powered and found a significant difference (p = 0.0012). Colored dots overlaid on the bar plots indicate the single-subject IOA average (3 trials per subject) for each subject. The legend on the right shows which colored dot corresponds with each subject. (C) Prosthesis knee torque calculated using inverse dynamics. Shown during sit-down with passive (gray) and powered (black) prostheses for subjects who did not use hands, from 0 to 120% of sit-down completion. Knee extension torque is negative. Lines indicate across-subject means and shading indicates standard error (N=7 subjects). (D) Relation between prosthesis knee position and prosthesis knee torque during sit-down with passive (gray) and powered (black) prostheses for subjects who did not use hands, from 0 to 100% of sit-down completion. Lines indicate across-subject means and shading indicates standard error (N=7 subjects). Circle indicates start of the movement (standing), square indicates the end of the movement (sitting)

### Statistics

We used paired t-tests to test for differences between powered and passive prosthetic conditions in the index of asymmetry, EMG peak, and EMG integral. Alpha was set at 0.01. We applied Bonferroni corrections for multiple comparisons to the EMG peak and integral to control for multiple comparisons of the EMG signal.

## Results

### Weight-bearing symmetry improved, and knee resistance increased

Weight-bearing symmetry, quantified using the index of asymmetry (IOA) of the vertical ground reaction force (GRF) averaged across time, improved when using the powered prosthesis compared to the passive prosthesis. The time-average of the IOA (N = 10 subjects) was closer to zero (more symmetric) when subjects used the powered prosthesis, and this improvement was observed during nearly the entire sit-down movement [Fig. 3A]. Our primary outcome measure, the across-subject mean IOA (i.e., the across-subject average of the single-subject IOA means) was significantly lower with the powered prosthesis than the prescribed passive prostheses (t(9) = 4.67, p = 0.0012). The across-subject IOA mean was 42.1% more symmetric when subjects sat down with the powered prosthesis (40.04 ± 4.42 during passive, 23.18 ± 5.19 during powered) [Fig. 3B]. Notably, every subject’s individual IOA average improved with the powered prosthesis [Fig. 3B, colored dots]. Corresponding with the improved weight-bearing symmetry, the powered prosthesis knee produced higher torques than the passive prosthesis – the powered prosthesis produced − 0.39 ± 0.06 Nm/kg peak torque, nearly twice the passive prosthesis knee peak torque (-0.24 ± 0.04 Nm/kg) [Fig. 3C]. The peak also occurred later in the movement with the powered prosthesis. This key advantage is highlighted by the torque-position curve: the measured peak torque with the powered prosthesis occurred at a higher measured knee flexion angle (68.70 ± 2.94 degrees) [Fig. 3D]. In comparison, the passive prosthesis knee torque peaked at 45.91 ± 2.13 degrees, much earlier in the movement [Fig. 3D].

**Fig. 4:**
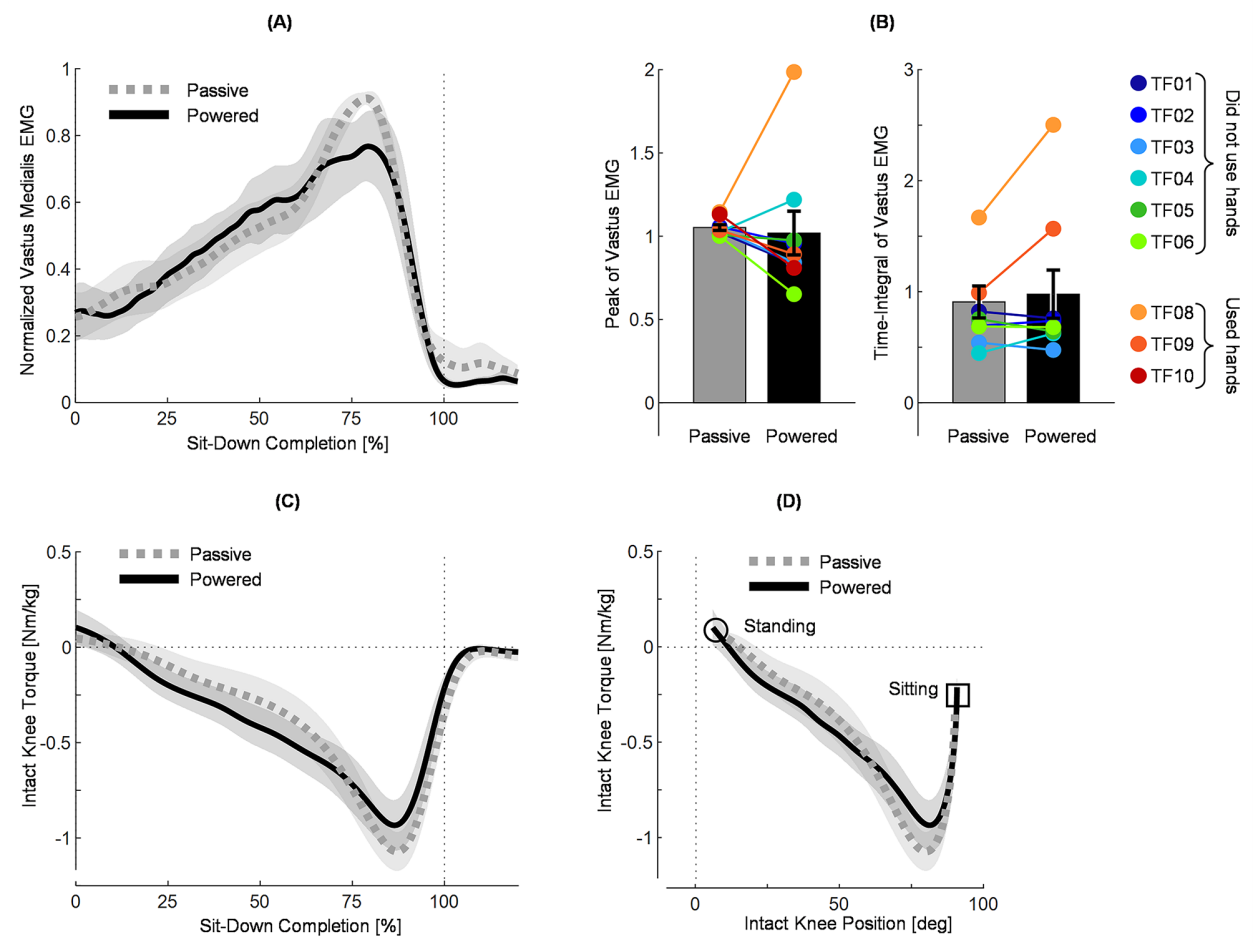
Muscle activity and torque from the intact leg. TF07 was removed from all parts of this figure (A-D) because of movement artifact in the vastus medialis EMG signal. (A) Normalized EMG from the intact-side vastus medialis muscle, shown from 0 to 120% of sit-down completion with passive (gray) and powered (black) prostheses. Lines indicate across-subject means and shading indicates standard error (N=9 subjects). (B) EMG peak (left) and EMG integral (right) between 0 and 100% of sit-down completion. Bar heights indicate across-subject means and error bars indicate standard errors (N=9 subjects). Colored dots overlaid on the bar plots indicate single-subject means (3 trials per subject). The legend on the right shows which colored dot corresponds with each subject. (C) Intact knee torque calculated using inverse dynamics. Shown during sit-down with passive (gray) and powered (black) prostheses for subjects who did not use hands (excluding TF07), from 0 to 120% of sit-down completion. Knee extension torque is negative. Lines indicate across-subject means and shading indicates standard error (N=6 subjects). (D) Relation between intact knee position and intact knee torque for all subjects who did not use hands (excluding TF07). Shown during sit-down with passive (gray) and powered (black) prostheses for subjects who did not use hands, from 0 to 100% of sit-down completion. Lines indicate across-subject means and shading indicates standard error (N=6 subjects). Circle indicates start of the movement (standing), square indicates the end of the movement (sitting)

### Intact-side quadriceps EMG and intact-side knee torques did not improve

The EMG from the intact vastus medialis muscle was similar for both prosthesis conditions. The intact EMG “shape” was slightly different between prosthesis conditions, but the metrics of intact knee muscle effort were not meaningfully different. The peak of the EMG changed from 1.06 ± 0.02 during passive to 1.03 ± 0.14 during powered, and the difference in peaks from passive to powered was not significant (paired t-test, t(8) = 0.2461, p = 0.8118, Bonferroni corrected p = 1). The time-integral of the EMG between 0 and 100% of intact-side sit-down completion changed from 0.99 ± 0.16 during passive to 0.97 ± 0.22 during powered, and this difference was not significant (paired t-test, t(8) = 0.1353, p = 0.8957, Bonferroni corrected p = 1). The intact knee torque also did not show large changes between conditions, peaking at -1.08 ± 0.09 Nm/kg during passive trials and − 0.93 ± 0.13 Nm/kg during powered trials. The intact knee during both passive and powered trials produced its peak torque later in the movement than either prosthetic knee, peaking at 78.92 ± 3.00 degrees during the passive trial and 80.85 ± 3.01 degrees during the powered trial.

### The powered prosthesis dissipated more energy than the passive prosthesis

**Fig. 5:**
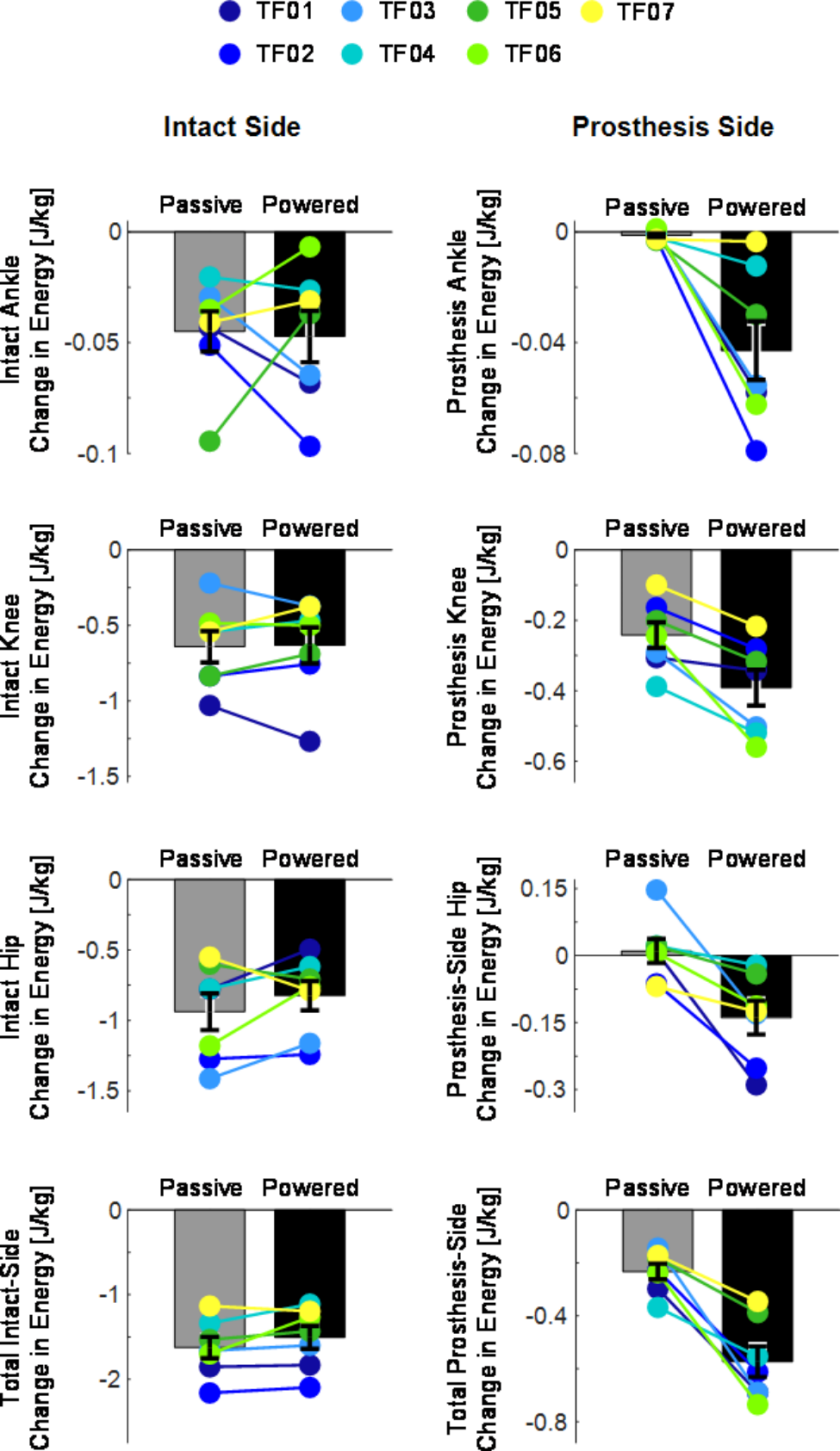
Energy injected (positive) or dissipated (negative) by each joint during the sit-down movement, calculated between 0 and 100% sit-down movement for all subjects that did not use hands during sit-down. The energy injected or dissipated by each joint is calculated as the area under a torque-position curve for that joint. Bar heights indicate across-subject means and error bars indicate standard error (N=7 subjects). Colored dots overlaid on the bar plots indicate the single-subject means (3 trials per subject) for each prosthesis condition. The legend at the top of the figure shows which color of dot corresponds with each subject

The energy change (injected or dissipated) by each joint, and the total energy dissipated by all of the joints of each lower limb, is shown in [Fig. 5]. The intact ankle energy was not consistently affected by prosthesis condition [Fig. 5, top row, left]. In contrast, the powered prosthesis ankle dissipated substantially more energy than the passive prosthesis ankles, and this effect was observed in every subject [Fig. 5, top row, right]. Similarly, the intact knee showed no trend, but the powered knee dissipated more energy than the passive knee for all subjects [Fig. 5, second row]. The intact hip dissipated slightly less energy during powered trials than passive for 5 of the 7 subjects, and the prosthesis-side hip dissipated more energy during powered trials than passive for all subjects [Fig. 5, third row]. The total sum of the energy dissipated by the intact ankle, knee, and hip joints decreased slightly during powered trials for 6 of 7 subjects, but the differences were very small [Fig. 5, bottom row, left]. The total sum of the energy dissipated by the prosthesis-side ankle, knee, and hip joints increased for all subjects during powered trials compared to passive [Fig. 5, bottom row, right]. The prosthesis-side joints dissipated 146% as much energy with the powered device compared to passive: 0.233 ± 0.0296 J/kg dissipated during passive trials, and 0.573 ± 0.0580 J/kg dissipated during powered trials. However, the intact side joints still dissipated more energy than the prosthesis side during both conditions: 1.625 ± 1.270 J/kg during passive, and 1.504 ± 0.136 J/kg during powered.

**Fig. 6:**
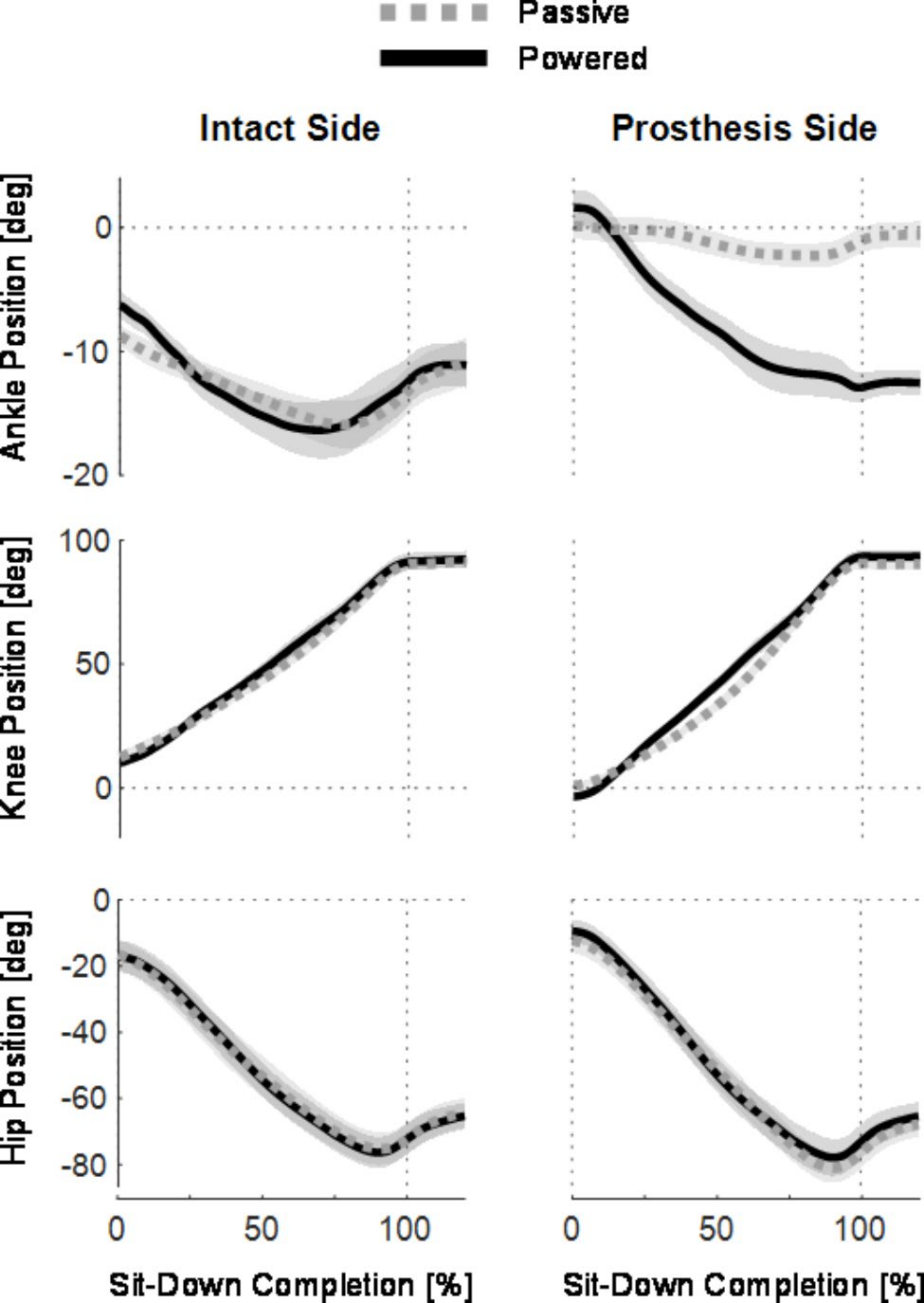
Lower limb joint positions plotted from 0 to 120% of sit-down completion with passive (gray) and powered (black) prostheses for all subjects. Lines indicate across-subject means and shading indicates standard error (N=10 subjects). Dotted vertical lines indicate 0% and 100% of sit-down completion. Dotted horizontal lines indicate 0 degrees. Plots in the left column show intact-side joint angles, and plots in the right column show prosthesis-side joint angles. The rows show ankle joint positions (top row), knee joint positions (middle row) and hip joint positions (bottom row)

### Lower limb kinematics and kinetics were affected by prosthesis conditions

The joint angles of the lower limb were very similar during powered and passive sit-down, with the notable exception of the prosthesis ankle joint. The prosthesis ankle joint range of motion was much smaller during the passive sit-down compared to powered, especially at the end of the movement [Fig. 6, top row, right]. Comparing initial joint angles, the intact side joints all begin the movement more flexed than the respective prosthetic side joints.

**Fig. 7:**
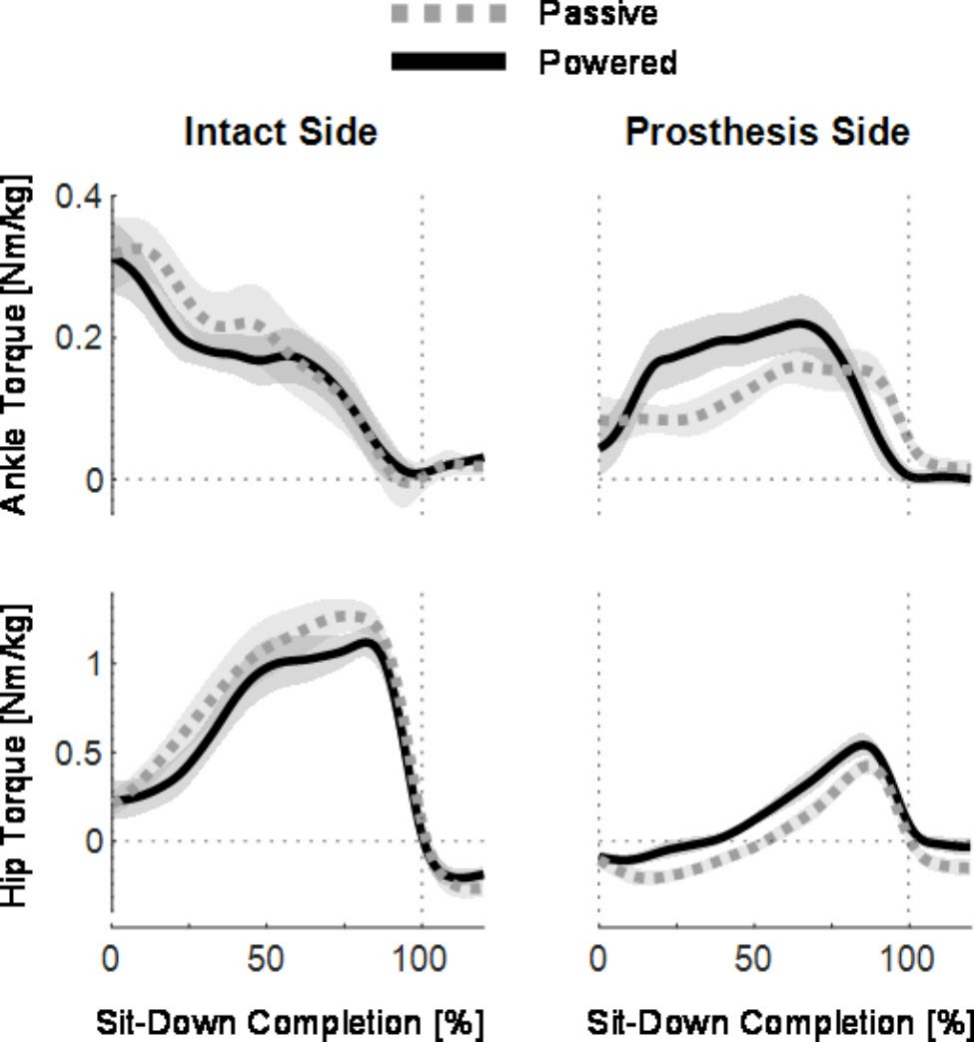
Ankle and hip joint torques plotted from 0 to 120% of sit-down completion with passive (gray) and powered (black) prostheses for subjects that did not use hands during sit-down. Lines indicate across-subject means and shading indicates standard error (N=7 subjects). Dotted vertical lines indicate 0% and 100% of sit-down completion. Dotted horizontal lines indicate 0 degrees. Plots in the left column show intact-side joint torques, and plots in the right column show prosthesis-side joint torques. The rows show ankle joint torques (top row) and hip joint torques (bottom row) calculated using inverse dynamics

The analysis of the ankle and hip joint torques shows notable differences between intact side torques and prosthesis side torques during passive and powered sit-down. The intact ankle plantarflexion torque (positive) was high at the beginning of the movement (at 0% of sit-down, 0.319 ± 0.052 Nm/kg during passive, 0.313 ± 0.052 Nm/kg during powered) and the torque decreased as the movement continued [Fig. 7, top row, left]. In comparison, both passive and powered prosthetic ankles began the movement with low levels of plantarflexion torque (at 0%, 0.084 ± 0.034 Nm/kg during passive, 0.044 ± 0.038 Nm/kg during powered) and increased during the sit-down movement for both prostheses [Fig. 7, top row, right]. Neither powered nor passive prosthetic ankle produced as much plantarflexion torque as the intact ankles at the beginning of the movement [Fig. 7, top row].

Similarly, the intact hip began the trial with some extension torque (positive) during both prosthesis conditions (at 0% of sit-down, 0.211 ± 0.072 Nm/kg during passive, 0.228 ± 0.109 Nm/kg during powered), and then produced high levels of peak extension torque at the end of sit-down [Fig. 7, bottom row]. In comparison, the prosthesis-side hip during both passive and powered trials produced a flexion torque (negative) at the beginning of the movement (at 0%, -0.106 ± 0.045 Nm/kg during passive, -0.090 ± 0.033 Nm/kg during powered). This early hip flexion torque was larger during the passive trial than the powered trial (peak flexion torque, -0.232 ± 0.048 Nm/kg during passive, -0.131 ± 0.035 Nm/kg during powered). In addition, the residual hip flexion torque continued until approximately 50% of sit-down completion during the passive trial, whereas the hip torque changed from flexion to extension earlier in the movement with the powered prosthesis. The peak hip joint extension torques on the intact and prosthesis-sides were slightly more symmetric during the powered trial than the passive trial – the intact hip peak torque decreased from 1.42 ± 0.10 Nm/kg during passive to 1.31 ± 0.08 Nm/kg during powered, and the prosthesis-side hip peak torque increased from 0.45 ± 0.06 Nm/kg during passive to 0.59 ± 0.05 Nm/kg during powered sit-down, a 30% increase [Fig. 7, bottom row]. However, even though the peak torque symmetry improved with the powered device, the prosthesis-side and intact-side torques were still very asymmetric.

Similar to the ankle and hip joints, the intact and prosthesis-side knee torques showed asymmetries during sit-down. The intact knee produced flexion torque during approximately the first 10% of the movement (peak flexion torque 0.127 ± 0.034 Nm/kg, or approximately 10.4 Nm during passive, and 0.153 ± 0.070 Nm/kg, or approximately 12.5 Nm during powered) and then began producing extension torque after approximately 10% of sit-down [Fig. 4C]. In comparison, the prosthetic knee produced approximately zero torque (neutral) at the beginning of movement (peak flexion torque 0.018 ± 0.008 Nm/kg, or approximately 1.47 Nm during passive, and 0.013 ± 0.010 Nm/kg, or approximately 1.06 Nm during powered) and then began providing resistive extension torque [Fig. 3C].

## Discussion

Microprocessor-controlled prostheses can support amputee users during net-negative energy tasks like stand-to-sit transitions by providing resistive torque at the joint level [12, 14, 19, 23, 26, 46]. However, these damping systems are limited in their ability to accurately control the resistive joint torque and cannot dissipate large amounts of energy, especially at large flexion angles [6–8, 47]. Using their embedded actuators, powered prostheses can more precisely control the joint torque while dissipating large amounts of energy, even at large knee flexion angles [33]. This study tested the hypothesis that a powered prosthesis can improve weight-bearing symmetry and reduce muscle effort during sit-down compared to passive prostheses by providing higher resistive torque at the prosthetic knee joint. Our experiments with ten individuals with above-knee amputations show that a powered prosthesis significantly improved weight-bearing symmetry but did not reduce the sound-side quadriceps muscle activations. The analysis of the joint kinetics shows that the powered knee and ankle joints provided substantially higher resistance compared to their passive counterparts, although the powered protheses still provided lower resistive torque than the intact leg. In this study, both passive and powered prostheses had net-negative knee mechanical energy, indicating energy dissipation. The powered prosthesis not only dissipated larger amounts of energy than passive, but is also capable of harnessing this dissipated electrical energy to recharge the battery [33]. Because the peak knee torque provided by the powered prosthesis was chosen based on the subjective preference of the participants and their ability to flex the knee without performing abnormal upper body movements, this result suggests that replicating the biomechanical function of the missing biological leg may not be possible with available controllers, prosthesis sockets, and prosthesis alignment techniques [48]. Thus, while powered protheses show meaningful improvements in clinically relevant outcomes such as weight bearing symmetry, they are still unable to replicate the biomechanical function of the missing biological leg. The kinetics, kinematics, and EMG analysis presented for the first time in this paper may enable further improvements in powered prosthesis outcomes.

Weight-bearing symmetry, measured using the index of asymmetry of the vertical ground reaction force, significantly (p < 0.01) improved by 42.1% when users sat down with the powered prosthesis compared to their passive prosthesis [Fig. 3B]. All subjects sat down more symmetrically when they used the powered prosthesis [Fig. 3B] and one subject (TF09) achieved negative index of asymmetry with the powered prosthesis, indicating that this subject put more weight on the prosthesis than on their intact leg on average. The powered prosthesis produced a peak knee torque that was nearly twice as high as the passive peak knee torque [Fig. 3D]. The powered knee peak torque also occurred later in the movement than the passive prosthesis peak torque (i.e., 46° vs. 69° during passive and powered, respectively) [Fig. 3D]. The knee position at peak torque is just as important, or even more important, than the magnitude of the peak torque during sit-down. As the knee flexes further, the gravity torque from the user’s mass produces increasingly large torques on the knees, and requires increasingly high support torques from the muscles, dampers, or motors of the knees to slow the user’s descent. The improvement we observed in weight-bearing symmetry seemed to be related to the increased peak knee torque produced by the powered prosthesis.

Although the powered prosthesis improved weight-bearing symmetry, subjects were still less symmetric than non-amputees. In this study, the index of weight-bearing asymmetry improved from 40 ± 4 with the passive prostheses to 23 ± 5 with the powered prosthesis, which was still higher (less symmetric) than the index of asymmetry reported for healthy controls, 6 ± 4 [44]. Moreover, the powered device did not match the peak torque or knee position at peak torque of the intact side – the intact knee peak torques were about twice as large, and occurred when the knee was 10° more flexed than the powered knee’s peak torque. Interestingly, at the end of the movement, the index of asymmetry increased (became less symmetric) after the powered knee torque had peaked and started to decrease, indicating that more support was still needed [Fig. 3A]. The increased asymmetry at the end of the movement may suggest that when the prosthesis torque began to wane, the users shifted their weight to their intact leg for support. These results suggest that the index of asymmetry was affected by the torque from the powered prosthesis occurring earlier and reaching a smaller peak compared to the intact knees. Thus, while the powered prosthesis significantly improved weight-bearing symmetry, we believe better results could be obtained by shifting the prosthesis peak knee torque to later in the movement when the knee is more flexed.

Although the powered prosthesis supported more of the users’ weight than the passive prostheses did, we did not observe significant reductions in intact-side muscle effort (p > 0.01 for peak and integral of EMG). Further analysis of the results show that the powered prosthesis decreased muscle effort for some subjects, and increased effort for others [Fig. 4B]. Moreover, the EMG envelopes with the powered and passive prosthesis were visibly different in shape. At the end of the movement, the EMG and intact side knee torque were lower with the powered prosthesis, probably because the powered prostheses provided substantially more support during the last part of the movement. In contrast, during the middle of the sit-down movement, the intact muscle effort and intact-side knee extension torques were slightly lower with the passive prosthesis [Fig. 4A, C]. A possible explanation for this result is that, when performing new movements or using new devices, users often co-contract their flexor and extensor muscles to stabilize their joints until they acclimate to the new movement or device [49]. Research has shown that individuals with amputations continue to adapt to a new device for weeks or even months [50, 51]. None of the subjects in this study had previous experience using the Utah Bionic Leg with the sit-down controller presented in this paper, but every subject used their own passive prosthesis every day. Therefore, it is possible that users were co-contracting to stabilize the sit-down movement due to their lack of training and experience with the powered prosthesis. We expect that if subjects were given more time to practice and acclimate to the powered prosthesis, they would be able to relax their intact side muscles more as they learned to fully trust the powered prosthesis.

Sit-down and stand-up are related movements, which are both essential for independent living. In our previous study of stand-up with a powered prosthesis, we observed similar improvements in weight-bearing symmetry [13]. During stand-up the index of asymmetry was measured at an extraction point which is not present during sit-down, and so different methods of extracting IOA metrics were used. However, improvements were observed regardless of extraction method. In our analysis of stand-up, we also observed significant improvement in muscle effort [13], which was not observed in this study of sit-down. Sitting down is scarier than standing up, because the movement requires a controlled fall into a chair which is behind the person – the endpoint is uncertain, and not easily visible. Anxiety may explain why subjects did not relax their muscles as much during sit-down compared to stand-up. Furthermore, during stand-up, a passive prosthesis produces zero assistive torque, acting as a simple hinge. During sit-down, a passive prosthesis produces some damping assistance. Therefore, during stand-up, there is more “room for improvement” compared to sit-down. The assistive torques during stand-up and sit-down were also different. In this study, we selected the highest level of assistance that each subject could sit down with, resulting in peak assistance levels that ranged from 0.15 to 0.6 Nm/kg. In our previous study of stand-up, every subject (N = 8) was able to stand up with assistance ranging from 0.2 to 1.6 Nm/kg [13].

### Limitations

Three subjects were unable to sit down with their passive prosthesis without using their hands, and if they needed hands during passive sit-down, they were asked to use hands during powered sit-down for consistency. Inverse dynamics is only valid if individual 6-axis force plates record each point-of-contact between the subject and the ground. We did not have force measurements on the chair, and so the inverse dynamics results (torque/energy) calculated for subjects who used their hands were invalid and excluded. The subjects who needed hands during sit-down were generally less strong and less mobile, and it is very difficult and demanding to sit down in a controlled way with only one intact leg. We believe that these individuals could have achieved sit-down with no hands given extensive training and practice to build confidence, but we believed that it was more representative to allow subjects to perform sit-down at their natural ability level. Most amputees do not sit down without using hands in their daily lives, because it is strenuous and potentially dangerous. However, this does not mean that only “strong” people can use the leg – quite the opposite. All three subjects who used hands had better weight-bearing symmetry when they used the powered prosthesis [Fig. 3, B]. In fact, the largest symmetry improvement was observed in TF09, a subject who used hands. Furthermore, TF09 is the only subject who was able to put more weight on the powered prosthesis [Fig. 3, B]. We made the determination to include subjects who could not sit down without hands for exactly this reason – weaker, elderly, and balance-impaired individuals are likely to benefit the most from powered prosthetics.

None of the subjects were able to sit down with a level of resistance that matched nonamputee peak knee torques [40]. When we tried to match non-amputee levels of resistive torque, subjects struggled to initiate or complete knee flexion without performing abnormal movements. Although this result may be explained, in part, by the effect of sockets and soft tissues, our biomechanical analysis suggests an alternative explanation. Nonamputees stand with slight flexion of the hip and knee, and initiate sit-down by relaxing their hip extensors, contracting their knee flexors slightly (~ 10 Nm) to bend the knee, and moving their center of gravity posterior to produce flexion torque at the knees [52]. In comparison, above-knee amputees normally stand with a more extended hip, which locks the prosthetic knee in extension as the prosthesis is aligned to be mechanically stable [47]. Thus, the residual hip flexors need to contract not only to flex the hip joint, but also to flex the prosthetic knee and dorsiflex the prosthetic ankle. Neither prosthesis produced meaningful knee flexion torques at the beginning of movement - the peak flexion torques were less than 1.5 Nm for both passive and powered. These small knee flexion torques are a direct result of the residual hip flexing while the foot is in contact with the ground, allowing the residual hip to produce interaction torques at the prosthetic joints – the passive prosthesis is not able to actively assist, and the powered prosthesis was not programmed to flex at the start of sit-down. To compensate for the lack of prosthesis knee flexion, we observed the residual hip producing substantial flexion torques to initiate the movement of both prostheses, whereas the intact hips never produced flexion torque [Fig. 7]. Therefore, subjects had to perform compensations to initiate sit-down with the powered prosthesis and passive prosthesis.

Several changes to the prosthesis controller could be implemented to reduce the observed compensations during movement initiation. The powered prosthetic knee did not flex synchronously with the residual hip, requiring the residual hip to work harder to initiate the movement of the prosthetic knee and ankle. This problem could be addressed, for example, by triggering powered prosthesis knee flexion torque when the user’s center of gravity shifts behind the prosthetic knee joint, which happens naturally at the beginning of sit-down [52]. Another potential solution would be to control the prosthesis knee using a neural signal, perhaps using EMG to control knee torque as in [12]. This could allow the user to control the powered prosthetic knee joint neurally, so that the hip and knee could move synchronously and initiate the sit-down movement using able-bodied movement strategies. However, further studies are necessary to assess the effectiveness of the discussed control changes.

Even if the controller delivered an ideal torque profile, it is likely that above-knee amputees would not reach full symmetry during sitting down. Prosthetics introduce inherent asymmetries to biomechanics of unilateral amputees. The prosthesis is connected to the user via a socket that suctions to the user’s residual limb. Within the socket, the residual femur is mobile within the soft tissues (skin and muscle) of the residual limb, which is known to negatively affect amputees’ movement control [53]. The residual femur must be braced against the socket in order to transfer any torques between the user and the prosthesis [53]. Another important factor is that the residual hip joint muscles are often altered during amputation surgery [48]. The muscles of the hip and thigh are rearranged to balance muscle forces at the hip to prevent contractures and to form a pad of muscles around the end of the bone to cushion the bone [48]. This surgery weakens the residual hip joint and reduces its range of motion [48]. Therefore, even if a powered prosthetic knee and ankle perfectly matched able-bodied biomechanics, the strength and movements of the residual hip will always be reduced and/or altered compared to the intact hip joint, impairing the user’s ability to transfer and control the torque from the prosthesis.

Prosthetic alignment introduces additional asymmetries. Prosthetists usually align above-knee prostheses so that the user is stable during quiet standing without expending excessive energy [47]. They do this by changing the angles and offsets of the knee and ankle interfaces until the ground reaction force vector passes through the center of the foot, and anterior to the prosthesis knee joint, during quiet standing. This alignment results in a knee extension torque which prevents the prosthesis from collapsing, and allows the residual hip to lock the knee by extending. However, this alignment also requires the residual hip to flex harder to unlock the prosthesis knee, as we observed in [Fig. 7]. Powered prosthetics are capable of mirroring able-bodied standing knee angles, and could allow for slight knee flexion during standing. However, in our experience, above-knee amputee users are very uncomfortable standing on a knee that is not locked, because it feels unnatural and unstable in comparison to standing with their at-home prosthesis. Therefore, most powered prosthetics are aligned the same way as passive prosthetics, to lock the knee during standing, which is more stable than able-bodied knee “alignment”. For these reasons, both passive and powered prosthetics introduce inherent kinetic and kinematic asymmetries which cannot be easily overcome by improved powered prosthetic control.

## Conclusions

In this study, we found that a powered knee-ankle prosthesis significantly improved weight-bearing symmetry during sit-down compared to passive prostheses for individuals with above-knee amputations. Weight-bearing symmetry improved significantly with the powered prosthesis, and every individual subject’s weight-bearing symmetry improved with the powered prosthesis. These improvements were associated with higher levels of knee torque from the powered prosthesis. However, the muscle effort from the intact-side leg did not improve with the powered prosthesis, nor did the intact-side knee torque change meaningfully. We observed that the powered prosthesis improved symmetry in nearly all results: weight-bearing symmetry, knee position at peak joint torque, peak joint torques, and joint energy dissipation. However, the powered prosthesis was unable to match intact-side values or healthy nonamputee values. Finally, we observed compensatory actions during sit-down with both the powered and passive prostheses. This is the first study to report full-body kinematics, inverse dynamic kinetics, EMG, and energy dissipation during sit-down with a powered knee-ankle prosthesis. Our results indicate that powered prosthetic devices can improve balance during sit-down for individuals with above-knee amputation, and that further improvements are likely possible by improving the powered prosthesis controller.

## Data Availability

All data and materials used in the preparation of this manuscript are available upon request.

## List of Abbreviations

3D: Three dimensional
deg: Degrees
EMG: Electromyography
GRF: Ground reaction force
IOA: Index of asymmetry
J: Joule
kg: Kilograms
m: Meter
mm: Millimeter
N (unit): Newton
N (statistics): Number of samples
Nm: Newton-meter
TF: Transfemoral
